# Encapsulation of a Neutral Molecule into a Cationic Clay Material: Structural Insight and Cytotoxicity of Resveratrol/Layered Double Hydroxide/BSA Nanocomposites

**DOI:** 10.3390/nano10010033

**Published:** 2019-12-21

**Authors:** Cristina Minnelli, Emiliano Laudadio, Roberta Galeazzi, Gianni Barucca, Valentina Notarstefano, Mattia Cantarini, Tatiana Armeni, Giovanna Mobbili

**Affiliations:** 1 Dipartimento di Scienze della Vita e dell’Ambiente (DISVA), Università Politecnica delle Marche, via Brecce Bianche, 60131 Ancona, Italy; c.minnelli@staff.univpm.it (C.M.); e.laudadio@staff.univpm.it (E.L.); r.galeazzi@univpm.it (R.G.); v.notarstefano@staff.univpm.it (V.N.); m.cantarini@staff.univpm.it (M.C.); 2 Dipartimento di Scienze e Ingegneria della Materia, dell’Ambiente e Urbanistica (SIMAU), Università Politecnica delle Marche, via Brecce Bianche, 60131 Ancona, Italy; g.barucca@staff.univpm.it; 3 Dipartimento Scienze Cliniche Specialistiche ed Odontostomatologiche, Università Politecnica delle Marche, via Brecce Bianche, 60131 Ancona, Italy; t.armeni@staff.univpm.it

**Keywords:** layered double hydroxide, resveratrol, drug delivery

## Abstract

Resveratrol (RES) is a stilbenoid polyphenol with interesting antitumor activity compromised by its poor solubility and bioavailability; thus, new approaches are necessary to improve its therapeutic effectiveness. In the present study, bovine serum albumin coated layered double hydroxide (LDH–BSA) was employed to encapsulate RES in order to overcome the above-mentioned usage limits. To evaluate the feasibility of neutral RES complexation with cationic LDH, we carried out molecular dynamics simulation in order to predict its structure and stability. In the supramolecular complex formed with LDH, RES disposes itself in the interlamellar region of LDH where it is stabilized by intermolecular interactions. The physico-chemical characteristics of the resulting nanocomplexes were studied by X-ray powder diffraction, transmission electron microscopy, and attenuated total reflection Fourier transform infrared spectroscopy. The encapsulation efficiency and drug release studies were also performed. The combined experimental and computational approach were highly effective in giving insight into the interaction mode of the neutral RES with the charged LDH. Finally, the nanohybrid’s anticancer ability was evaluated in human lung cancer cell line (A549) resulting in higher activity with respect to bare RES. Overall, the results showed that the nanocomposites are suitable for biomedical applications as delivery agents of RES.

## 1. Introduction

Resveratrol (RES) is a well-known biologically active stilbenoid polyphenol, present in numerous plant species including *Vitis vinifera*, produced in response to fungal infection, ultraviolet irradiation (UV) irradiation, and mechanical lesion. RES biological properties range from antioxidant to anti-inflammatory, neuroprotective, cardioprotective, antimicrobial, and anticancer activities [1,2]. In the RES chemical structure, two phenol rings are linked by an ethylene bridge, which can present both cis and trans configuration. The trans diastereomer (*trans*-3,5,4′-trihydroxystilbene) is prevalent and more active from a biological point of view; in particular, it is able to inhibit initiation, promotion, and progression stages in the carcinogenesis process. Furthermore, it can also display healthy effects because of its anti-inflammatory and antioxidant properties [2,3,4]. Despite the impressive potentiality due to the numerous molecular targets, RES is characterized by a very low bioavailability after oral administration (<1%) [5,6] due to its rapid metabolism and poor solubility in an aqueous environment (~0.13 mM) [5]. Among the approaches employed to increase RES bioavailability, pro-drug strategies, co-administration of biomolecules inhibiting metabolic enzymes, and nanotechnology techniques have been explored [7,8,9]. In this last field, nanocomposites incorporating both organic and inorganic constituents are a promising class of hybrid materials with unique properties that can be applied to catalysis, sensor development, microelectronics, energy packaging, and also as drug delivery systems [10,11,12]. They can be obtained by combining different nanomaterials or functionalizing nanomaterials with suitable molecules. 

**Figure 1:**
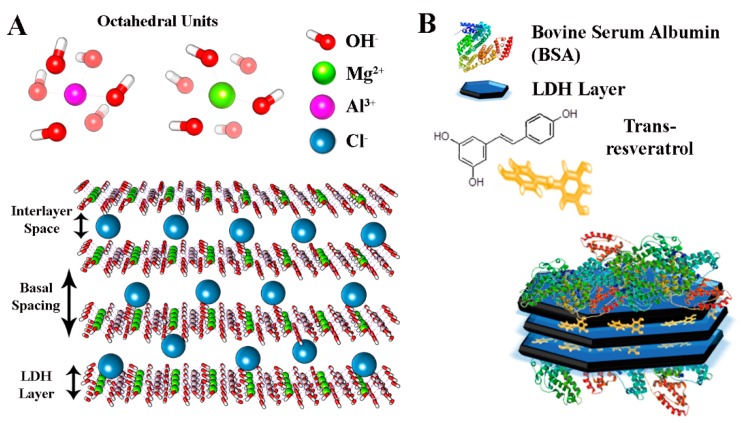
(**A**) In silico model of layered double hydroxides (LDH) used in this work and (**B**) representation of BSA-coated LDH nanocomposites.

Layered double hydroxides (LDH) are synthetic cationic clay materials with a brucite-like structure consisting of hydroxide layers where some of the divalent cations have been replaced by trivalent ions [12,13]. The resulting positively charged sheets interact with anions and hydrogen bonded water molecules present in the interlayer spacing, giving a material with a general composition formula of (M_1−*x*_^2+^ M*_x_*^3+^ (OH)_2_)*^x^*^+^(A^n−^)*_x/n_*·*m*H_2_O, where M^2+^, M^3+^, and A*^n^*^−^ represent a divalent cation, a trivalent metal cation, and an anion, respectively. The presence of hydroxyl groups on the surface and the intrinsic positive charge confer to LDH the potential to interact with polymeric molecules or other nanomaterials and generate three-dimensional nanocomposites with multiple functionalities [10,11,12,14,15,16,17,18]. This property together with their biocompatibility, availability, and possibility of modulating the structure through controllable ion-exchange processes, makes LDH suitable matrices for biomedical applications as delivery agents of anionic molecules. In this work, we produced nanoparticles with an LDH–Mg–Al core and a shell of bovine serum albumin (BSA) that confers particular colloidal stability to LDH nanoparticles (Figure 1).

The neutral trans RES was successfully intercalated into BSA coated LDH–Mg–Al, and the structural characteristics of the produced nanocomposites were determined by X-ray powder diffraction (XRPD), transmission electron microscopy (TEM), dynamic light scattering (DLS), and attenuated total reflection Fourier transform infrared spectroscopy (ATR–FTIR). The encapsulation efficiency and in vitro RES release were studied by gel filtration and dialysis methods both in the presence and absence of BSA, and several differences were observed. Before the synthesis and characterization of nanocomposites, computational experiments were used to assess the feasibility and stability of intercalating neutral polyphenols between positively charged LDH layers. In particular, molecular dynamic (MD) simulations have given information about the RES positioning specificity and configurations inside the nanocomposites showing a good correlation with experimental findings. Finally, to investigate the chemotherapeutic effect of these nanoparticles, the cytotoxicity of RES-loaded LDH–BSA nanohybrids was evaluated in vitro in human lung adenocarcinoma epithelial cell line (A549) and the results compared with that of bare RES.

## 2. Materials and Methods

### 2.1. Materials

Inorganic reagents including magnesium chloride hexahydrate (MgCl_2_·6H_2_O), aluminum chloride (AlCl_3_), and sodium hydroxide (NaOH), used for the layered double hydroxide (LDH) preparation, were purchased from Sigma Aldrich (St. Louis, MO, USA). RES, Sephadex G-50, 3-(4,5-dimethylthiazol-2-yl)-2,5-diphenyl-tetrazolium bromide (MTT), and all solvents were also obtained from Sigma Aldrich and used without further purification. Milli-Q ultrapure water (18.2 MΩ cm) was used throughout the study and adequately purged with N_2_ before use. The human lung adenocarcinoma epithelial cell line (A549) was obtained from American Type Culture Collection (ATCC), Italy office, Sesto San Giovanni, Italy.

### 2.2. Computational Methods

The crystal structure of LDH is based on the structure of brucite, with the Mg^2+^ cations partially substituted by Al^3+^ in the center of the edge sharing metal hydroxyl octahedral layers. In our computational model, we used the following formula:

Mg_3_Al(OH)_8_Cl^−^·*m*H_2_O. (1)

Since the amount of water molecules is strictly related to experimental conditions, for our model it was taken from data reported in literature [19]. Thus, a single LDH layer composed by 141 Mg(OH)_2_ and 47 Al(OH)_3_ molecules was built using the Macromodel MAESTRO suite [20]. A more extended model containing three layers was then generated, and the LDH basal spacing values were settled at 0.77 nm, according to literature data [21]. A periodic boundary condition (PBC) was then set up using a simulation box of (14.8 × 14.8 × 3.6) nm^3^ along x, y, and z axes respectively. The overall system containing 423 Mg(OH)_2_ and 141 Al(OH)_3_ molecules was then generated, adding 6345 flexible simple point-charge (SPC) water molecules and 141 Cl^−^ ions randomly distributed inside the simulation box to counterbalance the positive charge. Besides, another equivalent model was generated including RES loaded inside. Eight RES molecules, corresponding to a concentration of 0.4 mM, were introduced randomly oriented inside the layers. The structure energy of LDH/Cl^−^/H_2_O and LDH/RES/Cl^−^/H_2_O was minimized using a neighbor sourcing grid type. Then, this energy- and stress-minimized structure was used as starting point in molecular dynamic (MD) simulations. For both systems, an initial NVT-ensemble 30 ps of MD simulation was used for the equilibration, following an NPT-ensemble MD simulation 100 ns long at 300 K and 1 atm pressure. All MD simulations were performed using GROMACS 5.0.4 suite [22]. PBC and Ewald summation were used to consider the long range electrostatic interatomic interactions. The CLAYFF force field interatomic potentials [23] were used to describe the LDH layers, the SPC water molecules [24], and the Cl^−^ ions, while the AMBER force field [25] was used to describe the RES molecules. Visual molecular dynamics (VMD) [26] and Chimera [27] software were used for trajectory visualization and analyses, while Xmgrace (Grace 5.1.21 GNU public license, Cambridge, MA, USA) was used for generating plots.

### 2.3. Synthesis of LDH

The MgAl–Cl–LDH (LDH) were prepared using a co-precipitation method [13]. A solution of MgCl_2_·6H_2_O (0.67 g, 7 mmol) and AlCl_3_ (0.72 g, 3 mmol) in water (10 mL) was slowly added dropwise to a vigorously stirred solution (40 mL) containing NaOH (0.72 g, 18 mmol) at room temperature within 30 min. The LDH slurry was centrifuged at 500 g for 10 min and the pellet washed three times with deionized water (40 mL) until an almost neutral pH was reached. After adding deionized water (40 mL), the suspension was transferred into a stainless-steel autoclave with a Teflon lining and heated at 100 °C for 16 h. The final concentration of LDH suspension, obtained after lyophilization, was 10 mg mL^−1^. 

### 2.4. Pre-Coating LDH Nanoparticles with BSA

The preparation of LDH coated with bovine serum albumin (BSA) was performed as previously described with minor modification [17]. First, 12 mL of 10 mg mL^−1^ LDH suspension was dropwise added into 4 mL of 100 mg mL^−1^ BSA stock solution under vigorous stirring. The mixture was stirred at room temperature for 30 min to ensure protein adsorption. The resulting dispersion was centrifugated at 20,000× *g* for 20 min to separate the BSA–LDH from non-bonded BSA. The pellet of BSA–LDH was washed twice with deionized water to remove completely the unbonded BSA. The supernatants were used for the free BSA and LDH-associated BSA quantification. Then 1 mL of sample was diluted in acid solution (HCl, 1 M) to a final pH = 1 [8] to break the LDH that could eventually be present in the supernatant after the centrifugation, and the concentration of free BSA was determined spectrophotometrically at 280 nm using a UV–visible spectrophotometer (BioTek Synergy HT MicroPlate Reader Spectrophotometer, BioTek Instruments, Inc., Winooski, VT, USA) The calibration curve was plotted using free BSA dissolved in acid solution (HCl, pH = 1). Then, to indirectly determine the amount of LDH present in the LDH–BSA pellet, 5 mL of the collected supernatants containing free BSA and non-sedimented LDH were lyophilized. The amount of LDH in the supernatant was calculated by subtracting the calculated BSA amount from the total amount of material obtained after lyophilization.

### 2.5. Preparation of RES–LDHs and RES–BSA–LDHs Nanohybrids

The intercalation of RES molecules (abbreviated RES) into LDH and LDH–BSA host materials was obtained by mixing appropriate volumes of LDH and LDH–BSA suspension with a 40 mM solution of RES in ethanol under 1 h stirring. The final concentration was 4 mg mL^−1^ and 0.4 mM for LDH and RES, respectively. 

### 2.6. Nanoparticle Characterization

#### 2.6.1. Determination of Encapsulation Efficiency with Gel Filtration Method

LDH or LDH–BSA were separated from non-encapsulated RES by size exclusion chromatography. The disposable syringes (2.5 mL), packed with hydrated Sephadex G-25 resin (Sigma Aldrich, St. Louis, MO, USA) were placed in 15 mL plastic test tubes. After preconditioning with deionized water, 0.8 mL of LDH–RES and LDH–BSA–RES were gently added on the top of the syringes and centrifuged at 500 g for 10 min. Empty LDH and free RES were also used as controls. The eluates were collected at the bottom of the test tubes. To evaluate the encapsulation efficiency, 500 μL samples of purified and unpurified LDH/LDH–BSA were lysed by addition of acid solution (HCl, 1 M) to a final pH = 1 [8] and ethanol (1:1, v/v) to obtain a complete release and solubilization of the molecule, respectively. The RES concentration was measured at 307 nm by a UV–visible spectrophotometer (BioTek Synergy HT MicroPlate Reader Spectrophotometer, BioTek Instruments, Inc., Winooski, VT, USA), using a blank containing all the appropriate components except RES. The encapsulation efficiency (*EE*) of RES was calculated using the following formula (2):

*EE* (%) = 100 × (*A*_int_/*A*_total_) (2)

where *A*_total_ refers to the absorbance of RES measured in the unfiltered LDH/LDH–BSA, and *A*_int_ refers to the absorbance of the encapsulated molecule (which was the amount of RES measured inside purified LDH/LDH–BSA after lysis). All the experiments were repeated at least three times and measurements were run in triplicate. 

#### 2.6.2. Dynamic Light Scattering

The intensity-based diameter (Z-average) and the polydispersity index (PDI) of all systems (LDH, LDH–RES, LDH–BSA, and LDH–BSA–RES) were measured by dynamic light scattering (DLS) (Malvern Instruments GmbH, Marie-Curie-Straße 4/1, 71083 Herrenberg, Germania) and electrophoretic light scattering using a Malvern Zetasizer Nano ZS (Malvern Instruments GmbH, Marie-Curie-Straße 4/1, 71083 Herrenberg, Germania). An aliquot of every formulation was diluted at a final concentration of 3 mg mL^−1^ with ultra-purified water at the time of analyses. The colloidal stability of all systems was also studied in physiological conditions by mixing the samples with phosphate buffered saline (PBS) (pH, 7.4) supplemented or not with fetal bovine serum (FBS) (50% v/v) to reach a final concentration of 3 mg mL^−1^. All measurements were performed at 25 °C with a fixed angle of 173°. Particle size measurements and the polydispersity index were calculated from the autocorrelation function by cumulant analysis (Dispersion Technology Software V7.13 provided by Malvern Instruments). Zeta potentials were determined by the Zetasizer software (V7.13, provided by Malvern Instruments) from the electrophoretic mobility applying Henry’s equation and using Smoluchowski’s approximation. For all samples investigated, the data represent the average of at least three different autocorrelations carried out for each sample.

#### 2.6.3. X-ray Powder Diffraction

X-ray diffraction (XRD) measurements were performed in a Bruker D8 Advance (Bruker Nano GmbH, Berlin, Germany) diffractometer operating at *V* = 40 kV and *I* = 40 mA, with Cu-Kα radiation, in the angular range *2θ* = 5°–70°. For XRD analysis, samples were lyophilized and investigated in form of powders.

#### 2.6.4. Transmission Electron Microscopy

Transmission electron microscopy (TEM) analysis was performed by a Philips CM200 microscope (Philips, Amsterdam, The Netherlands) operating at 200 kV and equipped with a LaB6 filament. For TEM observations, samples, in the form of suspensions, were prepared using the following procedure. A drop of suspension was deposited on a commercial TEM grid covered with a thin carbon film. The grid was kept in air until complete evaporation of the solvent at room temperature.

#### 2.6.5. ATR–FTIR Spectroscopy

Infra-red (IR) analysis of LDH–RES, LDH–BSA, and LDH–BSA–RES systems and of their single components (LDH, BSA, and RES) was performed by using a PerkinElmer Spectrum GX1 spectrometer (PerkinElmer Inc., Waltham, MA, USA), equipped with a U-ATR (Universal- Attenuated Total Reflectance) accessory for the analysis of solid samples in reflectance mode. For each system/compound, 5 spectra were acquired in the 4000–450 cm^−1^ spectral range (64 scans, spectral resolution 4 cm^−1^). Before measuring each sample, the background spectrum was collected in the same experimental conditions. Raw IR spectra were converted in absorbance and vectors were normalized in the above defined spectral range; then, for each system/compound, the average absorbance spectrum was calculated (Opus 7.1 software package, Bruker Optics OPUS 7.1 software, Bruker Optics, Ettlingen, Germany).

#### 2.6.6. In Vitro Drug Release

The in vitro RES release from LDH and LDH–BSA was studied by the dialysis method after separation of untrapped RES by gel-filtration chromatography. Dialysis bags were soaked in PBS at room temperature for 24 h before use to remove the preservative, and then rinsed thoroughly in the same buffer solution. Then 8 mL of formulation was placed in the dialysis bag (12,000 MW cut off; Sigma-Aldrich) and dialyzed against 50 mL of release buffer (PBS, pH 7.4). Control bags, containing RES or BSA were prepared and dialyzed. The dialysis process was performed under stirring at 100 rpm at 37 °C and kept away from bright light. At scheduled intervals, 1 mL of the release medium was collected for analysis and the same volume of fresh PBS buffer was added immediately to maintain a constant release volume. The release medium was completely replaced with fresh buffer after 7 h to provide a continuous driving force for drug transport across the dialysis membrane. The cumulative amount of RES released was determined from the absorbance measured at 307 nm by a UV–visible spectrophotometer. The calibration curve was plotted using RES in PBS. The percentage released at each time point was expressed as a fraction of the total amount of RES. Drug release was monitored for 24 h. The data represent the average of at least five different analyses run in triplicate carried out for each sample.

### 2.7. Cell Cytotoxicity Assay

The human lung adenocarcinoma epithelial cell line (A549) was routinely maintained in 25 cm^2^ flasks in complete DMEM/F12 medium at 37 °C, 5% CO_2_, and 95% relative humidity. Complete DMEM/F12 medium was prepared by adding 10% (v/v) FBS, 2 mM glutamine, and 100 U/mL penicillin–streptomycin. The cell cultures were detached by trypsinization with 0.5% trypsin in PBS containing 0.025% EDTA and counted using trypan blue exclusion assay. For treatment, the cells were seeded in 24-well plates at 15 × 10^3^/well to reach 60% of confluence at 24 h, and then the medium was removed and replaced with 1 mL of fresh culture medium supplemented with free RES or LDH-based nanoformulations (0.15, 0.3, 0.6, 1.2, and 2.4 mg/mL of LDH) and incubated for 48 h. The free RES was added at concentrations equal to the RES content in the LDH-based formulations (RES, 10, 20, 40, 80, and 160 μM).

The number of metabolically active cells, and thus cell viability, was assessed by 3-(4,5-dimethylthiazol-2-yl)-2,5-diphenyltetrazolium bromide (MTT) assay [28]. At the time of analyses, the medium from each well was removed and replaced with fresh medium supplemented with MTT at a final concentration of 100 μg mL^−1^, and the A549 cells were incubated for 3 h at 37 °C in 5% CO_2_ atmosphere. Then 400 µL of DMSO were added to each well and to solubilize the purple formazan crystals formed from MTT reduction. The absorbance was read on a multiwell scanning microplate reader (BioTek Synergy HT MicroPlate Reader Spectrophotometer, BioTek Instruments Inc., Winooski, VT, USA) at 570 nm using the extraction buffer as a blank. The optical density in the control group (untreated cells) was considered as 100% viability. The relative cell viability (%) was calculated as (A570 of treated samples/A570 of untreated samples) × 100. Determinations were carried out in triplicate in each experiment and mean ± SD from five independent experiments was calculated.

## 3. Results and Discussion

### 3.1. LDH and RES Interactions: In Silico Simulations

**Figure 2:**
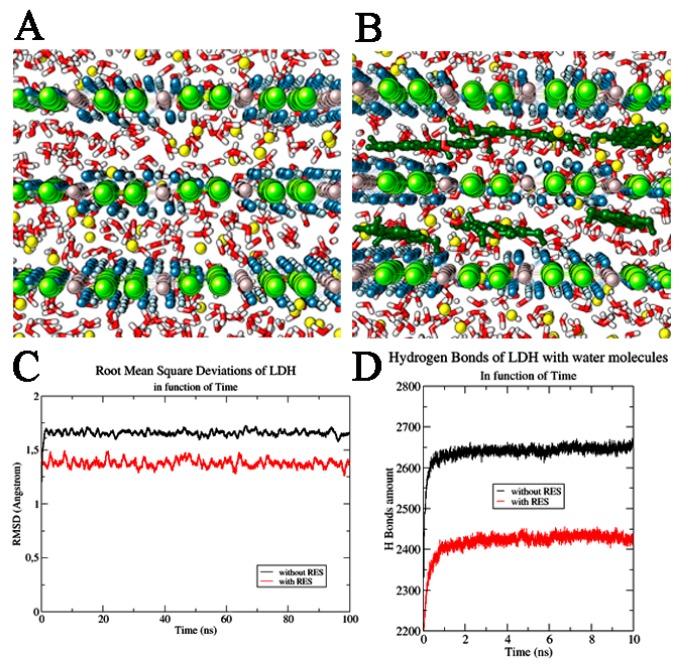
LDH systems. (**A**) LDH system without and (**B**) with resveratrol (RES) molecules. The water molecules are highlighted in red and white, Cl^−^ ions in yellow, Al^3+^ ions in grey, Mg^2+^ ions in green, and OH^−^ ions in blue and light blue. RES molecules are reported in dark green. (**C**) Root mean square deviation of LDH layers in function of time. (**D**) H Bond interactions of LDH with water molecules in function of time. We reported the system without RES (shown in black) and in the presence of RES (highlighted in red).

A preliminary computational study was performed in order to provide a molecular-scale insight into the MgAl–Cl–LDH interlayer structure and evaluate the potential intercalation of the neutral RES with the positive charged layers (Figure 2A,B). 

**Figure 3:**
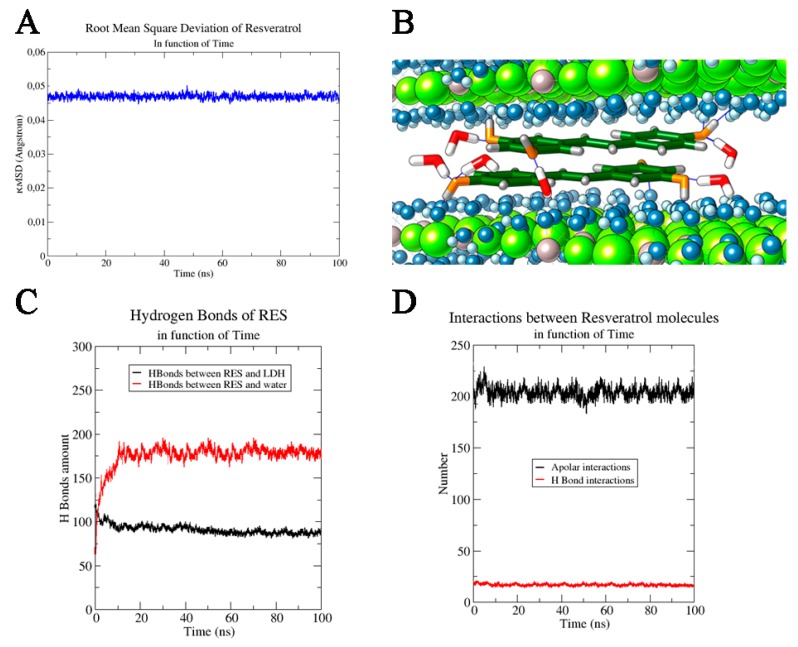
(**A**) Root mean square deviation of RES as a function of time and (**B**) RES molecules between two LDH layers. Cl^−^ ions are not shown for a major clarity of image, Al^3+^ ions and Mg^2+^ ions are reported in grey and in green respectively, OH^−^ ions are highlighted in blue (oxygen) and light blue (hydrogen), while only the water molecules (in red and white) directly involved in the H bond interactions with RES molecules are shown. RES molecules are reported in stick dark green, coloring by heteroatom. RES interactions in function of time: interactions of RES with (**C**) other species and with (**D**) itself.

The dynamic study of the pristine and RES-intercalated LDHs, carried out using NPT-ensemble MD simulations, revealed that both these systems are very stable in time as can be observed from the full MD trajectories (Root mean square deviation variation (ΔRMSD): LDH alone—Δ*RMSD* = 0.04 Å in last 20 ns, RES/LDH—Δ*RMSD* = 0.06 Å). In both cases, no changes in the dimension of the hydration phases were observed. In fact, the basal spacing of the hydrated LDH and LDH–RES presented a value very close to that set at the beginning of MD simulation (0.77 nm), with only slightly variations at the end of the trajectory. The distances between the water molecules and the Cl^−^ ions were also monitored along the MD simulation, and were maintained at almost constant (1.83 ± 0.01) Å. The average distance between the water molecules and the layer hydroxyls of LDH in both studied systems was also observed as being (1.7 ± 0.6) Å. All these distance ranges showed that the layers did not change their mutual interactions, confirming that RES insertion did not destabilize the LDH structural order, but in addition, it efficiently inserted deeply between the LDH layers. In fact, due to its π bonding delocalized system, RES had a quadrupole moment that allowed the polyphenolic molecules to efficiently interact with the positive charged LDH matrices, orienting its rings in a parallel mode with respect to the LDH planes; the molecule was further stabilized inside the bilayer by the formation of H-bonds with the water molecules. Trying to quantify these specific interactions, we observed in pure LDH an average of 2650 ± 20 hydrogen bond between LDH and water; the introduction of RES induced a significant decrease of these interactions, downsizing them to 2430 ± 20 (Figure 2D). RES molecules positioned between LDH layers physically hindered partially the water molecules’ direct interaction with the LDH layers. The high stability of RES between LDH layers was confirmed from the root mean square deviation (*RMSD*) values of the stilbenoid molecules along MD trajectories. For all 100 ns MD simulation, the RES *RMSD* value remained at (0.046 ± 0.002) Å (Figure 3A). 

This variation of *RMSD* (Δ*RMSD* < 0.001) along the MD simulation trajectory confirmed the stable structural conformation of the RES–LDH system; only an initial rearrangement (first ns) for RESs could be observed in the trajectory and it could be ascribed to the orientation of the aromatic rings parallel with respect to the LDH layers starting from its disordered random positioning in the input model (Figure 3B). Furthermore, the stability of RES–LDH complex was also underlined by the persistence of the intermolecular interactions (i.e., H-bonding) observed through all MD simulation times: (87 ± 5) H-bond interactions between RES and oxydryl of the LDH layers. Only a little decrease was observed in the first ns of MD simulation due to the above-mentioned reorientation of the aromatic ring in a parallel way with respect to the layer’s planes. On the contrary, considering the RESs’ H-bonds with water molecules, a drastically increase in the number of interactions was observed during the MD simulation, reaching a final average value of (190 ± 8) (last 10 ns) (Figure 3C). Another important contribute to the system stabilization was found in the intermolecular interactions between RES molecules. The presence of two aromatic rings conjugated to a double bond, and three hydroxyl groups conferred to RES on one side stiffness and on the other side allowed the stilbenoid molecules to interact efficiently with itself by both intermolecular H-bonding and face to face π–π stacking [29]. Since RES molecules oriented their aromatic rings parallel to the LDH planes, an additional stabilizing contribution arose from hydrophobic face to face π–π stacking interactions (Figure 3D). An average number of these apolar interactions was estimated to be 210 ± 20. A weak contribution in the hydrophilic interactions was found, and the number of RES–RES H-bond interactions was estimated to be 14 ± 2. This analysis of the molecular dynamics trajectories clearly showed that RES was more inclined to create hydrogen bonds with water and OH^−^ groups than with other RES molecules (Figure 3D). Furthermore, the favored interactions between RES molecules were hydrophobic, due to the planar RES structure and to the presence of aromatic rings and conjugated systems, which made the π–π stacking interactions extremely favored (Figure 3B).

In conclusion, the computational investigation assessed the high stability of RES between LDH layers, and the main forces of this stabilization were found in the hydrophilic interactions between the stilbenoid and both water molecules and the hydroxyl groups of LDH, and in the hydrophobic interactions between RES molecules. 

### 3.2. Preparation of RES Loaded LDH–BSA Nanocomposites: Stabilizing Effect of BSA

LDH samples were synthesized by a co-precipitation method, and the following intercalation reaction was performed by mixing the hydrotermically resized LDH suspension and the ethanolic RES solution. The colloidal stability was studied by dynamic and electrophoretic light scattering. In deionized water the systems appeared stable even after 1 month of preparation; the particles presented a mean diameter ranging from 100 to 120 nm, with a polydispersity lower than 0.3 (Table 1). 

When the LDH and LDH–RES formulations were added into a PBS solution, the mean diameter of LDH nanoparticles markedly increased; the positive charge of the LDH surface, in fact, caused the absorption of ions present in the salt solution with the consequent reduction of the zeta potential [30]. The diminished electrostatic repulsion determined the aggregation and precipitation phenomena observed in the formulation after 24 h of incubation. The aggregation observed in PBS could restrict their employment in a physiological environment. Since the addition of organic polymers or proteins can improve the colloidal stability of several types of nanoparticles [17,31,32], we used the negatively charged bovine serum albumin to obtain a precoating of LDH and prevent aggregation in a physiological buffer; the BSA high stability made it an ideal nanoparticle-stabilizing agent. LDH–BSA nanocomposites were synthesized by adding LDH suspension into BSA water solution (3:1, *w*/*w*). The unbonded BSA was quantified by spectrophotometry to exactly determine the mass ratio between BSA and LDH, which corresponded to 2:1, *w*/*w*. 

After these preliminary evaluations, the LDH–BSA–RES nanoparticles were prepared by direct complexation of LDH–BSA and RES to obtain after mixing a final concentration of 4 mg mL^−1^ and 0.4 mM, respectively. It is important to underline that the RES loading inside LDH–BSA was performed only after the complete removal of unbonded BSA to exclude the potential formation of BSA–RES complexes. The size distribution and zeta potential measures of all the analyzed formulations are showed in Table 1 at different time of incubation and conditions; the negatively charged BSA used to coat the positively charged LDH surface conferred high stability to LDH-based nanoparticles at all the conditions studied. The drastic decrease of positive zeta potential of LDH observed in the presence of PBS (from 42 mV in water to −15 mV in PBS) did not occur for the LDH–BSA system in the same conditions; the zeta potential was stabilized meanwhile at around neutral values. Due to the protein steric effect, the LDH–BSA (loaded or not with RES) did not aggregate despite their low zeta potential. The addition of RES in LDH–BSA or LDH did not significantly induce a change in particle size. 

### 3.3. Entrapment Efficiency of LDH and LDH–BSA

The encapsulation of RES inside LDH and LDH–BSA was determined by UV-spectroscopy, and the results are presented in Table 2.

For the LDH–RES we obtained about 70% of encapsulation efficiency of RES intercalation, which remained unchanged after 24 h and 1-month storage at room temperature in dark. In the presence of the BSA, we practically reached a complete encapsulation of RES (96% ± 2%), which decreased by 25% after 1 month of storage. Probably, the higher encapsulation efficiency observed was due to the interactions between the BSA surface and RES [33]; interestingly, the presence of the protein increased by about 26% the interaction of RES molecules with LDHs in the nanocomposite LDH–BSA–RES, and the same percentage of RES was lost after 1 month of preparation. 

### 3.4. Structural Insights from Nanocomposite

#### 3.4.1. X-ray Powder Diffraction

**Figure 4:**
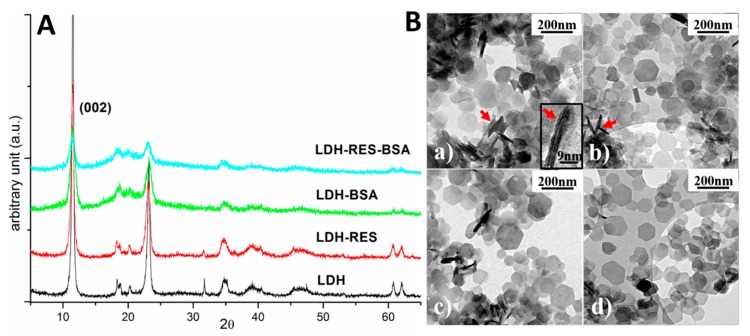
(**A**) X-ray diffraction spectra of LDH, LDH–RES, LDH–BSA, and LDH–BSA–RES samples. (**B**) TEM bright field images of: (**a**) LDH; (**b**) LDH–RES; (**c**) LDH–BSA; and (**d**) LDH–BSA–RES. Red arrows evidence some platelets perpendicular to the electron beam and one of these is magnified in the inset of (**a**).

X-ray diffraction (XRD) measurements of LDH, LDH–RES, LDH–BSA, and LDH–BSA–RES samples are shown in Figure 4A. 

LDH diffraction peaks can be attributed to a hexagonal phase having lattice parameters *a* = *b* = 0.305 nm and *c* = 1.55 nm, quite similar to those reported for magnesium aluminum chloride hydroxide hydrate (ICDD card no. 19-0748: *a* = *b* = 0.3070 nm and *c* = 1.550 nm). Comparing the four spectra, it is evident that all the visible diffraction peaks could be attributed to the pristine LDH sample. This result revealed that no other crystalline phases formed after adding resveratrol molecules or BSA proteins. Furthermore, the position of the diffraction peaks did not change. In particular, the peak associated with the family of lattice planes having Miller indexes (002) and corresponding to the distance among the layers of the layered double hydroxide (*d*_(002)_ = 0.77 nm) [21], was always in the same position revealing that the interlayer distance was independent of RES and BSA presence.

Since the BSA protein has a large dimensionality for a globular protein (40 Å × 40 Å × 140 Å), it was rather reasonable to think that it could not intercalate in the interlayer spacing of LDHs, as indeed was demonstrated by the X-ray results in which no expansion of the interlayer space was observed. On the other hand, the RES molecule has a smaller dimension and a possible penetration among the LDH (002) layers was plausible. However, considering XRD results, no variation was observed in the LDH crystals and this confirms the results obtained in MD simulations, where RES molecules were positioned in such a way as to not produce significant LDH deformation (Figure 3B).

#### 3.4.2. Transmission Electron Microscopy

Transmission electron microscopy (TEM) observations were performed in order to investigate the structure of the samples. Figure 4(Ba–Bd) shows typical TEM bright field images of LDH, LDH–RES, LDH–BSA, and LDH–BSA–RES, respectively All the samples were composed of thin platelets having a hexagonal shape and dimension ranging from 50 to 200 nm. The shape of the platelets reflects the hexagonal structure of the crystal lattice as revealed by XRD measurements. In the images, it is possible to observe some stacked platelets perpendicular to the electron beam (red arrows). Magnifying these perpendicular platelets, it is possible to observe fringes separated by about 1.5 nm (inset of Figure 4(Ba)), a distance compatible with the c-axis of the crystal lattice. Typically, platelets having thickness ranging from 1.5 to 4.5 nm were observed.

#### 3.4.3. Infrared Spectroscopy

**Figure 5:**
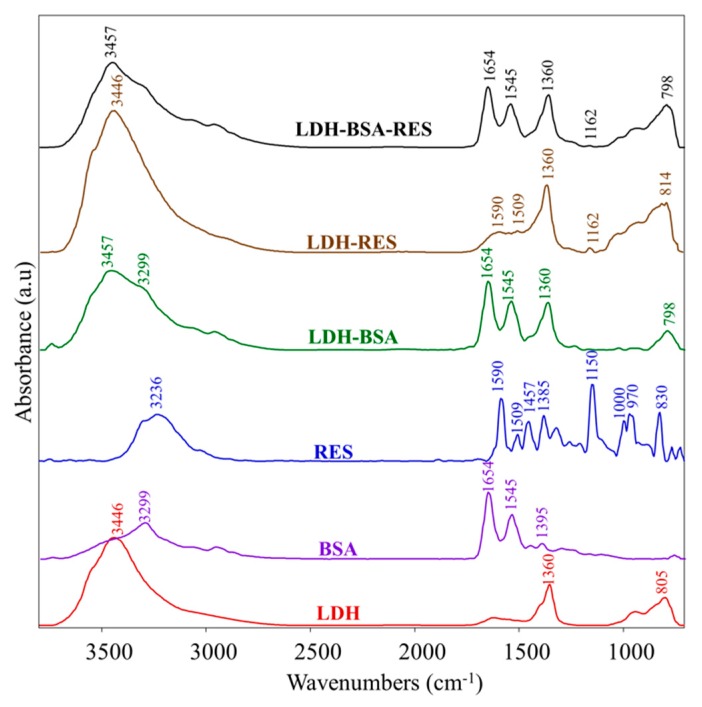
IR absorbance spectra of LDH–BSA, LDH–RES, and LDH–BSA–RES systems and LDH, BSA, and RES single components. Spectra are showed in the 3800–700 cm^−1^ spectral range; they are shifted along y-axis for better comprehension.

The vibrational analysis of LDH–BSA, LDH–RES, and LDH–BSA–RES systems was performed to detect structural modifications due to some interactions within the analyzed systems. In Figure 5, the IR absorbance spectra of LDH–BSA, LDH–RES, and LDH–BSA–RES systems and those of LDH, BSA, and RES single components are shown; in Table 3, the main vibrational modes of LDH, BSA, and RES are reported.

In the LDH–BSA system, the peak at 1360 cm^−1^ (νCO_3_^2−^) due to carbonate contamination in LDH and those of BSA at 3299 cm^−1^, 1654 cm^−1^, and 1545 cm^−1^ (respectively Amide A, Amide I, and Amide II bands, due to peptide bond) were detected, unchanged in the position [17,33]. Conversely, the absorptions of LDH at 3446 cm^−1^ and 805 cm^−1^ (due to the O-H bond) were shifted respectively to 3457 cm^−1^ and 798 cm^−1^, suggesting the formation of some interaction between LDH and BSA and hence confirming the protein absorption on the nanoparticles. In the LDH–RES system, the peaks attributed to RES at 1590 cm^−1^, 1509 cm^−1^, and 1162 cm^−1^ (νC–O), were found, the latter centered in RES at 1150 cm^−1^. In the LDH–BSA–RES system, the bands at 1654 cm^−1^ and 1545 cm^−1^ (attributed to BSA) and at 1360 cm^−1^ (due to carbonate contamination in LDH) were observed; in addition, the same shifts already found in LDH–BSA and LDH–RES were detected for the bands at 3457 cm^−1^, 1162 cm^−1^, and 798 cm^−1^, confirming the occurrence of a different H-bond network due to the interactions between RES and LDH oxydryl groups already observed in MD simulations [36]. 

### 3.5. In Vitro RES Release Behavior: Effect of BSA Coating

**Figure 6:**
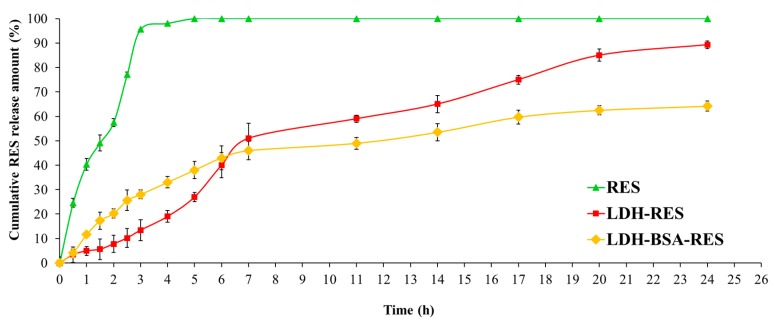
In vitro release of RES in PBS (pH 7.4) for LDH, LDH–BSA, and free drug solution. Values are expressed as mean ± SD; *n* = 3 independent experiments.

The RES release profiles from LDH and LDH–BSA were evaluated by dialysis method at 37 °C in PBS (pH, 7.4), and the results, compared with free RES behavior, are presented in Figure 6 as cumulative percentage release during 24 h. The diffusion of free RES through the dialysis membrane was more than 50% in the first 2 h and complete by 3 h, demonstrating that the dialysis membrane did not limit the release of RES. 

As shown in the Figure 6, the drug encapsulation inside LDH and LDH–BSA modified its release profile. LDH–RES had a gradual and biphasic release pattern with an early fast release of RES (51% of RES released after the first 7 h) followed by a relatively slower release, which resulted in almost complete release of RES in 24 h (89% of RES released). It is worth noticing that no burst phenomenon was observed at the beginning of release test; even the RES released from LDH particles took a much longer time than the release of other small drugs from MgAl–LDH at pH 7–8 [37,38]. This can be explained by the presence of stronger interactions of RES with LDH. 

LDH–BSA–RES showed a similar biphasic release pattern (Figure 6): 46% of RES was released in the first 7 h, as was similarly found in the LDH–RES system, but in this case, we did not observe a complete release (64% in 24 h). This difference in release rate was due to the protein LDH coating: the stabilizing effect of BSA may have decreased the amount of RES released in the buffer medium.

Several reports showed that the release of a drug over a long period of time led to a more efficient delivery and less side effects [39,40]: the biphasic and prolonged release profile resulting from a single dose of the LDH–BSA could benefit therapeutic treatment, as the initial fast release quickly allows establishment of a therapeutic dose, and the subsequent sustained release allows maintenance of this dose over a long period of time.

### 3.6. Cytotoxicity of LDH–BSA–RES

The potential antitumoral efficacy of the LDH–BSA–RES nanohybrids was studied by measuring the inhibition of cancer cell proliferation via MTT assay in lung cancer cells and comparing that with different concentrations of free RES (0–160 µM) and LDH–RES to investigate the role of BSA coating in cytotoxic characteristic of LDH–BSA–RES. Moreover, empty vehicles (LDH–BSA and LDH) were also tested to verify the potential toxicity of nanohybrids. 

**Figure 7:**
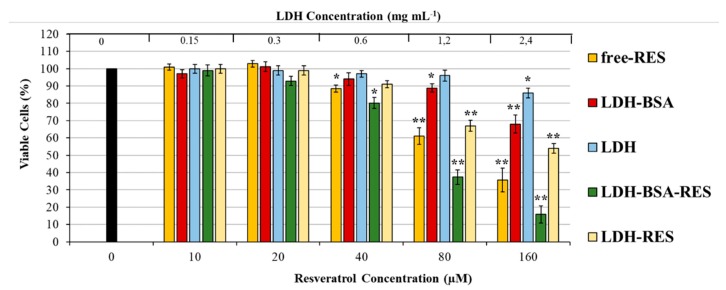
Cytotoxicity of free RES and RES loaded LDH–BSA and LDH in A549 cells. The cells were treated with increasing concentrations of LDH and LDH–BSA (loaded or non-loaded with RES) and free RES (at concentrations equal to the resveratrol content in the LDH–BSA and LDH formulation) for 48 h in complete culture medium. Cell viability was determined by MTT assay. Data are expressed as means ± S.D. of five independent experiments, each performed in triplicate, ** *p* < 0.001, * *p* < 0.05 difference from control, representing untreated cells.

As shown in Figure 7, free RES mediated growth inhibition of A549 cells in a dose-dependent manner as previously reported [41,42]. LDH–BSA had no significant effect in the 0–0.6 mg mL^−1^ range with slight cytotoxicity observed for 1.2 mg mL^−1^ (10% of cell death compared to non-treated cells). Interestingly, at 80 µM resveratrol concentration, the LDH–BSA–RES had a higher cell proliferation suppression efficiency compared to free RES (23% cell viability less than free RES-exposed cells, respectively). The improved colloidal stability, reached with BSA coating, allowed us to obtain about 29% more than with LDH–RES. The results showed the ability of nanocomposite to enhance the efficacy of resveratrol as anti-proliferative agents, which is essential future for cancer nanotherapy [43,44].

## 4. Conclusions

In this work LDH–BSA–RES nanocomposites were prepared and characterized for their potential use as drug delivery systems of the neutral and poorly water-soluble RES. The presence of BSA conferred high colloidal stability to the nanoparticles, as seen from the resulting DLS experiments. The potential anticancer ability of the nanohybrids was evaluated in human lung adenocarcinoma epithelial cell line (A549); the encapsulation improved the chemotherapeutic effectiveness of RES, and the cytotoxicity of the LDH–BSA–RES systems was higher with respect to bare RES. The combination of ATR–FTIR and molecular dynamics modeling approaches were highly effective in giving insight into the interaction mode of the neutral RES with the charged LDH. X-ray powder diffraction showed that the entrapment of RES in LDH–BSA nanocomposites did not produce significant deformations in the LDH lamellar phase. Overall, LDH–BSA can be considered as an effective inorganic host matrix for the entrapment and delivery of RES. 

## Figures and Tables

**Table 1:** Colloidal stability of BSA-coated LDH at different times and conditions of incubation.

Formulations	Time of Analyses	Size in Water ± SD (nm)	PDI ± SD (nm)	ζ-Potential ± SD (mV)	Size in PBS ± SD (nm)	PDI ± SD (nm)	ζ-Potential ± SD (mV)	Size in Serum ± SD (nm)	PDI ± SD (nm)
**LDH**	0 h	100.4 ± 0.9	0.221 ± 0.001	42.2 ± 0.3	3300 ± 700	0.6 ± 0.3	1.0 ± 0.7	131 ± 3	0.210 ± 0.006
	24 h	114 ± 2	0.225 ± 0.003		7000 ± 2000	0.3 ± 0.2		245 ± 3	0.270 ± 0.003
	1 month	127 ± 1	0.249 ± 0.002		-	-		-	-
**LDH-BSA**	0 h	171 ± 9	0.169 ± 0.005	−16 ± 4	170 ± 9	0.16 ± 0.01	−10 ± 3	171 ± 9	0.17 ± 0.03
	24 h	169 ± 7	0.158 ± 0.003		167 ± 4	0.140 ± 0.003		167 ± 4	0.140 ± 0.003
	1 month	182 ± 9	0.258 ± 0.004		-	-		-	-
**LDH-RES**	0 h	122 ± 6	0.20 ± 0.02	33 ± 2	600 ± 100	0.7 ± 0.3	1.0 ± 0.7	146 ± 8	0.202 ± 0.009
	24 h	124 ± 5	0.22 ± 0.02		5000 ± 1000	0.8 ± 0.3		238 ± 4	0.243 ± 0.003
	1 month	118 ± 8	0.36 ± 0.02		-	-		-	-
**LDH-BSA-RES**	0 h	171 ± 4	0.172 ± 0.009	−16 ± 2	169 ± 8	0.15 ± 0.02	−17 ± 4	169 ± 4	0.17 ± 0.01
	24 h	173 ± 8	0.160 ± 0.008		173 ± 7	0.180 ± 0.016		166 ± 7	0.03 ± 0.01
	1 month	210 ± 10	-		-	-		-	-

**Table 2:** Encapsulation efficiency of LDH and LDH–BSA loaded with RES.

Formulations	Time of Analyses	Encapsulation Efficiency ± SD (%)
**LDH-RES**	0 h	70 ± 4
	24 h	72 ± 3
	1 month	66 ± 6
**LDH-BSA-RES**	0 h	99 ± 1
	24 h	96 ± 2
	1 month	71 ± 6

**Table 3:** Main vibrational modes of LDH, BSA, and RES.

System/Compound	IR Band	Vibrational Mode	Reference
**LDH**	3446 cm^−1^	ν O-H, stretching vibration of O-H bonds	[17,33]
	1360 cm^−1^	ν CO_3_^=^, stretching vibration of carbonate groups	
	805 cm^−1^	δ O-H, bending vibration of O-H bonds	
**BSA**	3299 cm^−1^	δ N-H, bending vibration of N-H bonds (Amide A band)	[34]
	1654 cm^−1^, 1545 cm^−1^	ν C=O, ν C-N, and δ N-H, vibrational modes of peptide bond (Amide I and II bands, respectively)	
	1395 cm^−1^	δ C-H, bending vibrations of protein side-chains	
**RES**	3226 cm^−1^	ν O-H, stretching vibration of O-H bonds	[35]
	1590 cm^−1^, 1509 cm^−1^, 1000 cm^−1^	ring stretching vibrations	
	1457 cm^−1^, 1385 cm^−1^, 970 cm^−1^, 830 cm^−1^	δ C-H, in-plane and out-of-plane bending vibrations of C-H bonds	
	1150 cm^−1^	ν CC, stretching vibration of vinylidene moiety	
